# Multiple Paternity in Garter Snakes With Evolutionarily Divergent Life Histories

**DOI:** 10.1093/jhered/esab043

**Published:** 2021-08-05

**Authors:** Eric J Gangloff, Megan B Manes, Tonia S Schwartz, Kylie A Robert, Natalie Huebschman, Anne M Bronikowski

**Affiliations:** 1 Department of Ecology, Evolution, and Organismal Biology, Iowa State University, Ames, IA, USA; 2 Department of Zoology, Ohio Wesleyan University, Delaware, OH, USA; 3 Department of Biological Sciences, Auburn University, Auburn, AL, USA; 4 Department of Ecology, Environment and Evolution, La Trobe University, Bundoora, VIC, Australia

**Keywords:** life-history evolution, multiple mating, reproductive skew, sexual selection, *Thamnophis elegans*

## Abstract

Many animal species exhibit multiple paternity, defined as multiple males genetically contributing to a single female reproductive event, such as a clutch or litter. Although this phenomenon is well documented across a broad range of taxa, the underlying causes and consequences remain poorly understood. For example, it is unclear how multiple paternity correlates with life-history strategies. Furthermore, males and females may differ in mating strategies and these patterns may shift with ecological context and life-history variation. Here, we take advantage of natural life-history variation in garter snakes (*Thamnophis elegans*) to address these questions in a robust field setting where populations have diverged along a slow-to-fast life-history continuum. We determine both female (observed) and male (using molecular markers) reproductive success in replicate populations of 2 life-history strategies. We find that despite dramatic differences in annual female reproductive output: 1) females of both life-history ecotypes average 1.5 sires per litter and equivalent proportions of multiply-sired litters, whereas 2) males from the slow-living ecotype experience greater reproductive skew and greater variance in reproductive success relative to males from the fast-living ecotype males despite having equivalent average reproductive success. Together, these results indicate strong intrasexual competition among males, particularly in the fast-paced life-history ecotype. We discuss these results in the context of competing hypotheses for multiple paternity related to population density, resource variability, and life-history strategy.

Reproduction requires energetic investment by both females (e.g., number and size of offspring) and males (e.g., territorial defense, mate guarding, sperm size, and quality; reviewed in Schwarzkopf and Shine 1991). Understanding energetic trade-offs in these components of reproduction is the foundation of life-history theory (Roff 1992, Stearns 1992). Therefore, differences in reproductive strategy and cost between the sexes are informative traits to study when unraveling the mechanisms behind life-history evolution (Glaudas et al. 2020, reviewed in Wedell et al. 2006). This is because differential investment in reproduction among individuals in a population can result in some individuals having disproportionately more offspring than others (known as reproductive skew; Clutton-Brock 1989). This increased variance in reproductive success can be either between sexes or among individuals of the same sex and may result in trade-offs with other life-history characters (e.g., a negative relationship between reproductive effort and longevity).

Multiple paternity—when more than one male contributes genetically to a single reproductive bout of a single female—is widespread across the animal kingdom (Taylor et al., 2014). The causes and consequences at the individual and population levels are myriad and likely depend on ecological and life-history contexts. For example, multiple paternity can result either in greater reproductive skew or in a more uniform distribution of males contributing to the next generation. In some vertebrate populations, reproductive skew is exaggerated in promiscuous mating systems due to variance in the occurrence of multiple paternity (e.g., in swordtail fish, Luo et al. 2005) or a small number of males dominating a harem (e.g., in elephant seals, Hoelzel 1999). In other contexts, multiple paternity may reduce—instead of increase—variance in male reproductive success (Pearse and Anderson 2009). At the individual level, fitness benefits of multiple mating can be direct. For example, female Texas field crickets (*Gryllus texensis*) gain benefits to immune function (Worthington and Kelly 2016) and reproductive output (Worthington et al. 2015) from multiple matings. Fitness benefits of multiple mating may also be indirect and extend across generations (Jennions and Petrie 2000). For example, in dark-eyed juncos (*Junco hyemalis*), offspring produced from extrapair offspring enjoy higher reproductive success compared with offspring produced by a social pair (Gerlach et al. 2012). And in marbled salamanders, offspring from multiply-sired clutches have higher survival to metamorphosis (Croshaw et al. 2017). Differences in the benefits of multiple matings between males and females can lead to sexual selection and increased reproductive skew, either within or between sexes. Increased reproductive skew can, in turn, decrease the effective population size, and ultimately the ability of a population to respond to selection due to increased inbreeding (Charlesworth 2009). Thus, understanding the specific context and outcomes of multiple paternity sheds light on the interplay between reproductive life-history traits, reproductive strategy, and their evolution.

In snakes, multiple paternity has been identified in nearly every taxa tested and is therefore likely the ancestral reproductive system in snakes (reviewed in Rivas and Burghardt 2005; Uller and Olsson 2008; Voris et al. 2008; Wusterbarth et al. 2010; Jellen and Aldridge 2011; Meister et al. 2012). Past work in snake species has examined the potential drivers of differences among populations in the frequency of multiple paternity (Garner et al. 2002; Prosser et al. 2002; Friesen, Kerns, et al. 2014; Stedman et al. 2016), but we still lack a thorough understanding of how mating systems, life histories, and sexual conflict interact to shape variation in multiple paternity and consequent male reproductive success. In resource-rich environments where most reproductively mature females can dedicate resources to offspring, male–male competition (e.g., combat or sperm competition) may result in relatively fewer males, compared with females, contributing to the next generation (reviewed in Friesen et al. 2020), for examples in snakes, Prosser et al. 2002; Blouin-Demers et al. 2005). In contrast, unpredictable environments and those with limited resources may prompt females to mate with multiple males to increase the genetic diversity of her offspring and thus increase the number of males siring offspring in a population, a strategy generally referred to as bet-hedging (Yasui 1998, 2001; Calsbeek et al. 2007; Wapstra and Olsson 2014). Resource limitation could also decrease the number of males reproducing by increasing variance in male condition and via increased male-male competition.

The populations of western terrestrial garter snakes (*Thamnophis elegans*) surrounding Eagle Lake in Lassen County, CA, offer a well-established study system to test for differences in multiple paternity and reproductive skew in the context of the evolution of life-history strategies. These discrete populations of snakes are dichotomous in habitat type, found either around the rocky shore of Eagle Lake, with constant food and relatively high levels of predation, or in higher elevation mountain meadows, with fluctuating levels of food and water that are dependent upon annual snow melt (Miller et al. 2011; Sparkman et al. 2013; Gangloff et al. 2020). Divergence in life-history characteristics between the 2 types of habitats has resulted in 2 distinct life-history ecotypes of *T. elegans* (Bronikowski and Arnold 1999; Addis et al. 2017), which differ in morphology (Manier et al., 2007), behavior (Gangloff et al. 2017), and physiology (Robert and Bronikowski 2010; Palacios et al., 2012; Schwartz and Bronikowski 2013; Gangloff et al., 2015). Most relevant to this study are the striking differences in reproduction between the 2 ecotypes (Sparkman et al. 2007). In lakeshore habitats, snakes having a faster pace-of-life (“L-fast” ecotype), grow quickly, achieve larger asymptotic adult sizes, reach sexual maturity earlier, and reproduce more often and with larger litters relative to slower pace-of-life meadow snakes (“M-slow” ecotype). Mating occurs primarily in spring, but can occur throughout the active summer season (personal observation). Females gestate over the summer and give birth to young on a single day in autumn. As with other species of garter snake, the females are capital breeders and depend on the stored resources from the previous year for reproduction in a current year (Rossman et al. 1986; Gregory and Skebo 1998; Madsen and Shine 1999). During gestation, females must actively thermoregulate to maintain optimal body temperature for embryonic development (Arnold and Peterson 2002; O’Donnell and Arnold 2005) and many stop eating midway through gestation (Bronikowski and Arnold 1999). This is evident in energetic trade-offs, for example pregnant females display lower T-cell proliferative ability and lower counts of white blood cells than non-pregnant females (Palacios and Bronikowski 2017). Thus, reproduction is costly for females because of energetic allocations to vitellogenesis and developing embryos, reduced foraging and ingestion capacity, impaired locomotor ability (Seigel et al. 1987; Van Dyke and Beaupre 2011), and lower adaptive immunity.

*Thamnophis elegans* is nonterritorial with a polygynandrous mating system and no parental care, and, therefore, no post-parturition cost of provisioning on either sex. Therefore, we expect levels of multiple mating to be high if costs of mating for females are low. If females are not exerting choice for sires (used here to include both mate choice and/or cryptic choice via in utero sperm selection), we would expect the level of multiple paternity to also be high. The only previous study of multiple paternity in this species detected paternity from up to 3 males in a single litter (*N* = 6 litters; Garner and Larsen 2005). If encounter rates are equivalent between ecotypes and there is no female choice, we predict increased multiple paternity in the L-fast ecotype due to their larger litter sizes simply because numerically, more ovulated eggs yield increased potential for fertilization. That this is not generally seen in reptiles (Uller and Olsson 2008; Jellen and Aldridge 2011) suggests that females are exerting some control over number of sires (either through pre- or postcopulatory mechanisms. As well, a positive correlation between litter size and number of sires could further arise from sexual selection for males to mate with larger, and therefore more fecund, females (Garner et al. 2002; Becher and Magurran 2004; Garner and Larsen 2005; Neff et al. 2008; Jellen and Aldridge 2011), thus increasing the number of sires contributing to the largest litters. Differences in litter sizes but not levels of multiple paternity would suggest that females with smaller litters are employing strategies to maintain levels of multiple paternity despite fewer opportunities for fertilization (i.e., fewer offspring). Using these garter snake populations with divergent life-history ecotypes, we specifically test for differences in the occurrence of multiple paternity, the average number of fathers per litter, male reproductive success, and female reproductive success. To quantify the consequences of these life-history strategies, we compare 3 measures of reproductive skew: between sexes within a population, among individuals within each sex, and among sires within litters. We provide an empirical test of association between life-history traits and mating systems in natural populations.

## Materials and Methods

### Populations and DNA Sampling

**Figure 1.**
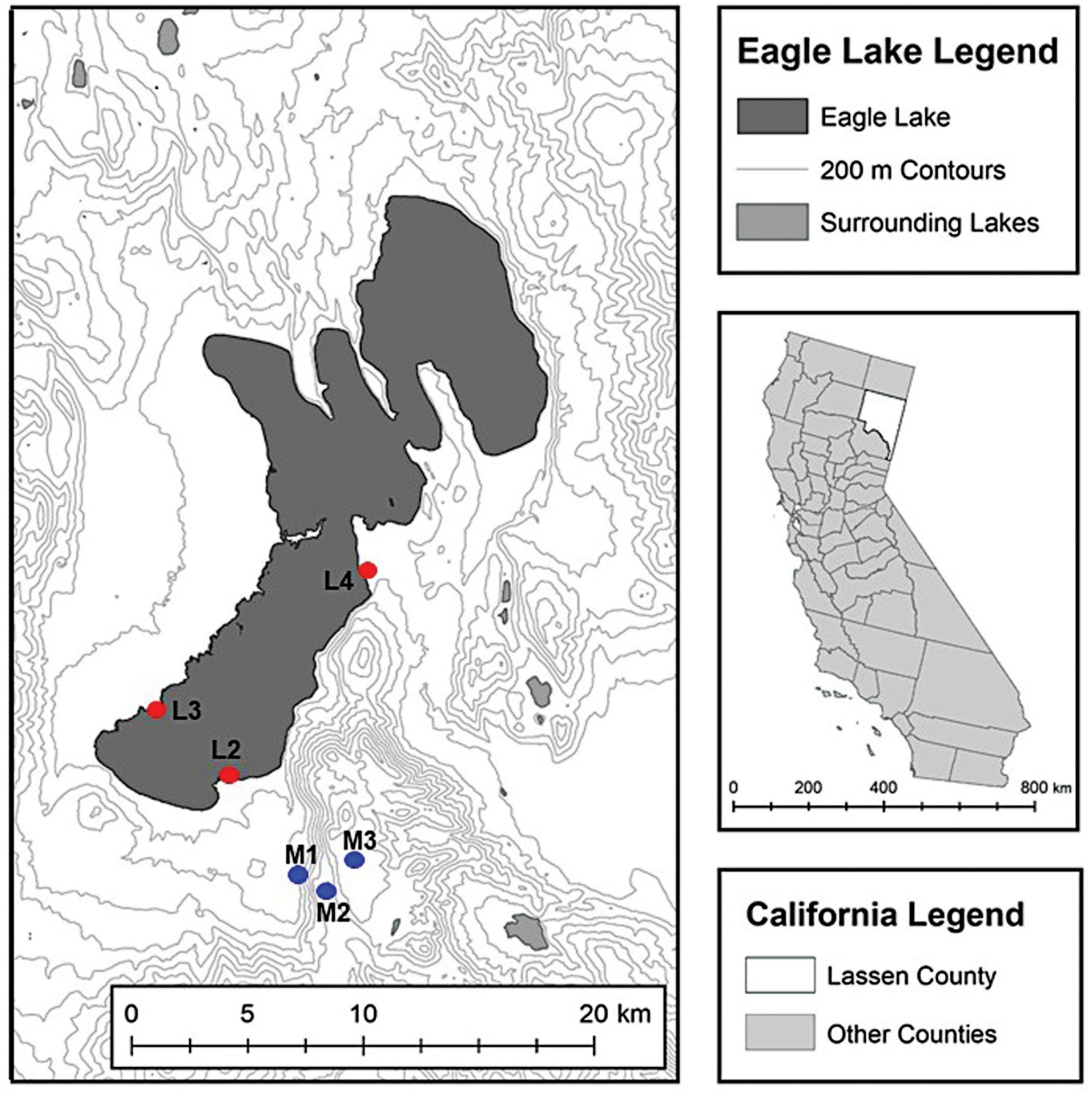
Map of population locations of *Thamnophis elegans* around Eagle Lake, Lassen County, CA, with M and L indicating replicate Meadow-slow (blue) and Lakeshore-fast (red) populations. Numbering from Bronikowski and Arnold 1999. Map created by Katie Fetting using ArcGIS 10.3 (ESRI, Redlands CA).

We collected tissue samples from adult snakes from 3 L-fast and 3 M-slow populations surrounding Eagle Lake in Lassen County, CA, from 2006 to 2008 (Figure 1, Table 1). Snakes were sexed, weighed, and measured (snout-to-vent length [SVL] in mm) at the time of capture. In 2006, 56 gravid females (L-fast: *N* = 30, M-slow: *N* = 26) were returned to Iowa State University and their litters were born in captivity. Neonates (*N* = 463 live-born, 12 still-born) were sexed, weighed, and measured (SVL) within 24 h of birth. Additionally, muscle tissue from the tail tip was sampled from each neonate for genetic analyses. Populations are designated by letter (M for M-slow, L for L-fast) and number combinations previously used in Bronikowski and Arnold (1999), with the addition of an L-fast population L4 (Figure 1, Table 1). In addition to these gravid females, we collected a tissue sample from additional adult males and females from each of the 6 populations to estimate population-level genotype frequencies (*N* = 213 additional males and females).

**Table 1.** Sample sizes and genetic diversity measures computed from the 8 microsatellite loci in *Thamnophis elegans*

Habitat	Sample sizes					Diversity measures						
Population	N_Adults_	N_Males_	N_Females_	N_Litters_	N_Offspring_	GD (SD)	H_O_ (SD)	N_A_ (SD)	P_A_	F_IS_	PIC (SD)	P_EXC_
L-fast												
Total/Avg	104	33	71	30	323	0.71 (0.05)	0.71 (0.04)	6.2 (2.2)	6		0.68 (1.5)	
L2	56	27	29	13	154	0.66 (0.04)	0.65 (0.02)	7.0 (2.4)	1	0.008	0.69 (1.4)	0.003
L3	7	0	7	4	26	0.77 (0.05)	0.74 (0.06)	5.0 (1.4)	1	0.045	0.67 (1.7)	0.004
L4	41	6	35	13	143	0.71 (0.04)	0.69 (0.03)	6.6 (2.4)	4	0.026	0.67 (1.4)	0.004
M-slow												
Total/Avg	165	87	78	26	140	0.71 (0.05)	0.71 (0.02)	7.8 (2.9)	4		0.64 (1.4)	
M1	63	39	24	8	52	0.71 (0.04)	0.69 (0.02)	7.9 (2.8)	1	0.019	0.65 (1.4)	0.003
M2	52	25	27	9	33	0.70 (0.05)	0.68 (0.02)	7.1 (3.0)	2	0.026	0.61 (1.4)	0.003
M3	50	23	27	9	55	0.72 (0.05)	0.70 (0.02)	7.9 (2.4)	1	0.031	0.67 (1.4)	0.003
Overall Total/average	269	120	149	56	453	0.71 (0.05)	0.71 (0.03)	7.0 (2.6)	10		0.66 (1.5)	

GD, Nei’s unbiased genetic diversity; *H*_O_, observed heterozygosity; *N*_A_, average number of alleles; *P*_A_, number of private alleles; *F*_IS_, system of mating inbreeding coefficient; PIC, polymorphic information content, a measure of the information content of the loci for paternity analysis related to expected heterozygosity; *P*_EXC_, is the probability of not excluding a random candidate male as the father if the mother genotype is known (calculated in cervus).

### Genotyping

We purified DNA from tissue samples using a standard salt extraction method modified from Sunnucks and Hales (1996). Purified DNA was diluted to 25 ng/µL to achieve consistent genotyping results. Eight highly variable microsatellite loci reported in previous publications (Garner et al. 2004; Manier and Arnold 2005; McCracken et al. 1999; Prosser et al. 1999) were modified slightly for this study (Table 2). An M13-tail was added to one primer from each locus to allow tagging with M13 fluorescently labeled primers (Boutin-Ganache et al. 2001). The loci were organized into 3 multiplexes for amplification, using a Type-it Microsatellite PCR kit (Qiagen, Venlo, The Netherlands). All loci were amplified in 7-µL reactions using 25 ng DNA, 0.03–0.65 nM of each locus-specific reverse and forward primer, 0.5–0.6 nM of the M13 primer labeled with a fluorescent dye, and 3 mM MgCl_2_ (in Type-it Multiplex PCR Master Mix, Qiagen). Amplification was performed with a Bio-Rad iCycler (Bio-Rad Laboratories, Hercules, CA) at 95 °C for 5 min to denature, and then a cycle repeated 30 times of 95 °C for 30 s, 57 °C for 1 min 30 s, and 72 °C for 30 s, and lastly an extension of 30 min at 60 °C. After amplification, 0.5 µL of each sample from each multiplex (1.5-µL total PCR product per sample) was mixed with 8.5-µL H_2_O to create a 1:20 dilution. 1.5 µL of each of these diluted samples was electrophoresed at the Iowa State University DNA Facility using the ABI 3100 Genetic Analyzer (Applied Biosystems, Foster City, CA). To minimize scoring biases and errors, we used GeneMapper (Applied Biosystems) to define allelic bins. The electropherograms were scored automatically by GeneMapper and subsequently verified or corrected by eye, independently by 2 researchers (M.B.M., T.S.S.).

**Table 2.** Properties of the microsatellite loci used in this study

Locus	Multiplex	Final concentration (nM)	Allele size (bp)	Total alleles	Avg H_O_	Avg H_E_	Avg PIC	Null alleles	Reference
*Nsμ10*	3	F = 0.03 R = 0.45	140–162	7	0.626	0.618	0.54	0	Prosser et al. (1999)
*Nsμ2*	2	F = 0.03 R = 0.45	181–185	5	0.612	0.630	0.55	0	Prosser et al. (1999)
*Nsμ3*	3	F = 0.03 R = 0.35	167–219	14	0.849	0.859	0.82	0	Prosser et al. (1999)
*TS10*	1	F = 0.03 R = 0.35	147–187	11	0.815	0.814	0.77	0	Manier and Arnold (2005)
*TS2*	2	F = 0.03 R = 0.15	136–169	10	0.669	0.678	0.63	1	McCracken et al. (1999)
*Te1Ca18*	1	F=0.03 R = 0.28	96–131	11	0.633	0.615	0.57	0	Garner et al. (2004)
*Te1Ca2*	1	F = 0.03 R = 0.50	232–266	13	0.760	0.781	0.74	0	Garner et al. (2004)
*Te1Ca3* ^a^	2	F = 0.04 R = 0.65	91–123	9	0.579	0.692	0.63	3	Garner et al. (2004)
Total/average				80	0.693	0.711	0.66	0.5	

For the concentration used, F refers to the forward primer, and R refers to the reverse primer. *H*_O_, observed heterozygosity; *H*_E_, expected heterozygosity; PIC, polymorphic information content. See text for details.

^a^Excluded from paternity analysis due to rejection of Hardy–Weinberg equilibrium and presence of null alleles (see text for details).

### Loci Verification and Population Genetic Measures

The genotypes of field-caught adults (*N* = 269, including the *N* = 56 pregnant females) were analyzed in the program MicroChecker (Van Oosterhout et al. 2004) to estimate null allele frequencies and to test for large allele dropout and the Wahlund effect. Pairwise estimates of linkage disequilibrium were calculated in GenePop (Raymond and Rousset 1995). We tested for deviations from Hardy–Weinberg equilibrium (HWE) for each locus by population combination in Arlequin v.3.11 (Excoffier et al. 2005) using an Exact test with Markov chain lengths of 10 000 with 1000 dememorization steps. Significance values were corrected for multiple testing using a sequential Bonferroni correction (Rice 1989). The *Te1Ca3* locus was dropped from the subsequent population structure and paternity analyses due to the presence of null alleles and significant deviation from HWE (see Results).

For each population, estimates of genetic diversity were averaged over all loci. These estimates included observed and expected heterozygosities (Avg *H*_O_ and Avg *H*_E_, respectively), unbiased genetic diversity (Nei 1987), the size range of the alleles in base pairs (bp), average number of alleles, total number of alleles, and number of private alleles. Polymorphic information content (PIC; Bolstein et al. 1980) and paternity exclusion power were calculated in Cervus 3.0 (Kalinowski et al. 2007). We used Arlequin (Excoffier et al. 2005) to calculate pairwise *F*_ST_ values to estimate genetic distance among the populations and Fstat (Goudet 2001) to estimate the inbreeding coefficient (*F*_IS_).

### Paternity Analysis

We successfully assigned paternity to 411 of the 463 (89%) of the live-born offspring. The majority of offspring were genotyped from each litter (minimum 58% of offspring in a given litter, mean 86.3%). Restricting analyses to litters where 100% of babies were sampled (*N* = 22 litters) does not qualitatively change results (analyses not shown), therefore we present the results with this 58% cutoff. The minimum number of fathers that contributed to each litter was determined using Colony (Wang 2004). This program estimates the likelihood that offspring are related to each other (i.e., are siblings), as well as the likelihood that any of the genotyped adults are the mothers and fathers of the offspring. For litters where not all offspring were successfully genotyped, the actual number of fathers may be higher. The data set included genotypes of known mother/offspring relationships and genotypes for adult males serving as potential fathers, without any designation of relatedness. Each population was analyzed separately using population-specific allele frequencies calculated from the adult samples in that population. We estimated rates of errors (i.e., null alleles, mutations, genotyping error) and mismatches between mother and offspring alleles using MicroChecker. Using the allele frequencies, as well as the known mother–offspring relationships, Colony generated expected genotypes of the males contributing to each offspring in a litter with a 95% confidence level. Colony settings were as follows: polygamous mating system, dioecious and diploid species, medium run length, full-likelihood analysis method, medium likelihood precision, and no sibship size prior. To assess the accuracy of our results and to identify the effects of different error rates on the number of fathers assigned to a litter, simulations were conducted in Colony utilizing varying genotyping error rates (0–2%), as well as different run lengths (“medium” to “very long”) and precision levels (“medium” to “high”). Our paternity assignments were robust to these variations.

### Multiple Paternity and Reproductive Success

We used a chi-square test to assess differences in the levels of multiple paternity between ecotypes (binary response variable: Y/N). Because the possible number of fathers contributing to a litter ranged from 1 to 4 in this study (see Results), to test for differences in the number of males contributing to each litter between ecotype we utilized a multiple ordinal logistic regression, including in the model population nested within ecotype and the number of offspring sampled within each litter. To account for differences in litter size between ecotypes (L-fast > M-slow), we conducted a permutation analysis and restricted the litter size for L-fast dams to the median litter size of the smaller M-slow litters (median = 5). For each L-fast dam, we made pseudo-litters by sampling her offspring with replacement to fill this litter of 5, keeping dam and sire associations, and thus making no assumptions about sperm precedence or competition. We repeated this procedure for the sampled population 999 times and calculated the average number of sires for each truncated L-fast litter. We then compared the distribution of paternity in the populations of pseudo-litters with the observed values. If the observed value of sires per litter differs from the number of sires of truncated litters, this would suggest that females may actively employ strategies to alter levels of multiple paternity beyond a sampling effect due to differences in litter sizes.

Reproductive success was measured in females as total live litter size and was measured in males as the number of offspring each male sired. Male reproductive success is necessarily limited to the offspring sampled and may therefore not represent the total number of offspring in the population fathered by a single male. We used ANCOVAs to test the effects of ecotype and population nested within ecotype on female and male reproductive success. The analysis of female reproductive success also included the covariate of body size (SVL). Statistical analyses were performed in SAS 9.1 (SAS Institute, Cary, NC).

### Estimates of Reproductive Skew

To evaluate how the reproductive traits within each ecotype could affect the distribution of reproductive success among different groups of individuals, we calculated 3 estimates of reproductive skew within each population or ecotype: (1) To test for differences in the distributions of reproductive success, we employed a Kolmogorov–Smirnov test (Massey 1951) at 3 levels: between males and females within ecotypes, among males between ecotypes, and among females between ecotypes. (2) To test for skew of reproductive success among breeders within a sex we used an Index of Variability (“I-V”; Araki et al. 2007), the variance in reproductive success among individuals of the same sex divided by the mean reproductive success for that sex in a population. Based on the expected Poisson distribution, a value of one indicates that reproductive success was evenly spread among the individuals within that sex in that population, whereas an inflated value indicates one or more members of that sex had disproportionately higher reproductive success. (3) We used a skew estimate from Neff et al. (2008) to assess the distribution of sires contributing to each litter (within-litter skew) as an indicator of sperm selection and/or cryptic female choice. Values significantly different from zero, as assessed with a one-tailed *t*-test, indicate a skew among sires contributing to each litter.

## Results

### Loci Verification and Population Genetic Measures

Of the 168 pairwise within-population loci comparisons, 12 were found to have linkage disequilibrium between pairs of loci, but only one (between *TS10* and *Te1Ca18* in M2 population) was significant after sequential Bonferroni correction. Because these loci only showed linkage in one population, we assume it is an artifact and not actually due to physical linkage. All loci in all populations were in HWE except for *Te1Ca3*, which had significantly reduced heterozygosity relative to the expected in both L4 and M2 populations. MicroChecker predicted the presence of null alleles (i.e., an allele that is not detectable due to mutation) in loci within 4 populations used in this study, with 3 of the 4 instances attributed to *Te1Ca3*. Therefore, we did not include the *Te1Ca3* locus in the analyses of parentage in Colony or population structure in Arlequin. The *TS2* locus showed evidence of null alleles in only one population (M1), and this error estimate was incorporated into analysis for this population at this locus in Colony.

The populations had similar measurements of genetic diversity, inbreeding coefficients (*F*_IS_), and PIC; thus, the loci were equally suitable for determining paternity across all the populations (Table 2). The global *F*_ST_ for these populations was 0.068 (*P* < 0.0001); of the 15 pairwise *F*_ST_ values, 12 were significant at *P* < 0.05 and 11 were still significant after sequential Bonferroni corrections (Table 3). This genetic differentiation among populations suggests it is unlikely that males from one population could be potential fathers in other populations. This is supported by our mark-recapture data over the past 40 years, where we have captured only a few female migrants and no male migrants (A.M.B., unpublished data).

**Table 3.** Population genetic structure among *Thamnophis elegans* populations

	L2	L3	L4	M1	M2
L3	0.001				
L4	0.019*	0.00			
M1	0.076*	0.046*	0.075*		
M2	0.067*	0.042*	0.072*	0.003	
M3	0.065*	0.039*	0.069*	0.008*	0.007

Pairwise *F*_ST_ values (calculated using 7 loci) below diagonal. Asterisk indicate significant values after sequential Bonferroni correction. See Supplementary Table S1 and Supplementary Figure S1 for additional structure information.

### Paternity Analysis

We genotyped an average of 86.3% of the offspring from the 56 litters (range: 58–100% genotyped, combining both ecotypes). Multiple paternity was detected in all 6 populations. The maximum number of fathers identified in a single litter by Colony was 4, which was found in 2 litters, one M-slow (population M2) and one L-fast (population L4). A total of 68 sires contributed to these 56 litters, as identified by unique paternal genotypes contributing to offspring within each litter. Only 6 sires had known identities from being caught in the field (i.e., 6 of the 120 field-caught males included in the Colony analysis). Of the 411 analyzed offspring, 34 were assigned to these 6 males with known identities.

### Multiple Paternity and Reproductive Success

**Figure 2.**
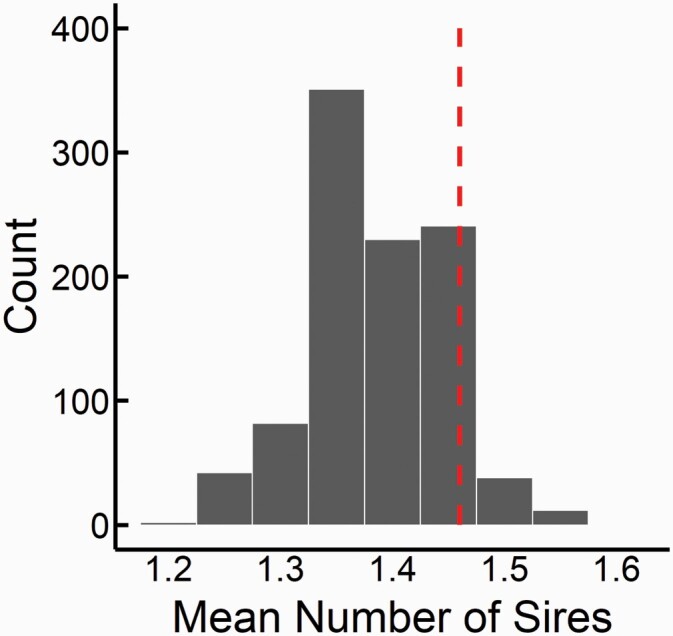
Distribution of average number of sires per litter for L-fast populations from simulations restricting L-fast litter sizes to M-slow median litter size of 5. Frequency is the number of times the mean number of sires was observed out of 999 iterations. The red line indicates the observed average number of sires for M-slow litters (1.46 sires per litter). The mean number of simulated L-fast fathers was not significantly different from observed M-slow values (*P* = 0.136).

The number of multiply-sired litters did not differ between the ecotypes: 50% of litters in L-fast, 38% of litters in M-slow ($\chi_{1}^{2}$ = 0.36, *P* = 0.55; Table 4). The mean (± SD) number of sires for each ecotype was 1.60 (range: 1–4) sires per litter for L-fast populations and 1.46 (range: 1–4) sires per litter for M-slow populations, and ecotypes did not differ (multiple ordinal logistic regression: Wald’s $\chi_{1}^{2}$ = 0.024, *P* = 0.88; Tables 4 and 5). Neither the sampled litter size nor maternal size affected the number of sires in each litter and the lack of difference between ecotype holds if we remove these covariates from the model. The mean number of sires per litter within individual populations ranged from 1.25 to 1.77, and did not differ significantly among populations (multiple ordinal logistic regression: Wald’s $\chi_{1}^{2}$ = 3.63, *P* = 0.46; Tables 4 and 5). The permutations restricting the large L-fast litters to smaller M-slow sizes showed that, L-fast litters would not differ in number of sires form observed M-slow litters (i.e., 1.39 vs. 1.46, *P* = 0.136; Figure 2).

**Table 4.** Components of reproductive skew among populations of *Thamnophis elegans* for males and females

Ecotype	Population	Number of breeders	Reproductive success^a^	% multiply-sired litters^a^	I-V^a^	Skew within litter	Observed sires per litter	Operational sex ratio
**L-fast**								
	**Dams**	30						
	L2	13	11.84	38%	0.91		1.46	0.62
	L3	4	6.50	50%	1.49		1.50	0.86
	L4	13	11.00	62%	0.45		1.77	0.25
	**Dam Avg.**		10.77	50%	0.98		1.60	0.55
	**Sires**	39				0.12***		
	L2	16	8.69		4.20	0.07*		
	L3	5	4.20		2.55	0.06		
	L4	18	6.78		2.72	0.17**		
	**Sires Avg.**		7.23		3.59			
**M-slow**								
	**Dams**	26						
	M1	8	6.50	25%	1.10		1.25	0.40
	M2	9	3.67	44%	0.27		1.67	0.68
	M3	9	6.11	44%	0.22		1.44	0.97
	**Dam Avg.**		5.38	38%	0.82		1.46	0.71
	**Sires**	29				0.07**		
	M1	8	6.00		0.81	0.10		
	M2	10	3.00		0.89	0.04		
	M3	11	4.64		0.96	0.08*		
	**Sires Avg.**		4.45		1.15			

Number of breeders in the study for each population; least-squares means of average male and female reproductive success (see text for details); percent of litters with multiple paternity; Index of Variability (I-V) among breeding individuals of the same sex in a population; skew among sires within a litter; average number of observed number of sires; operational sex ratio (values less than 1.0 are female biased). Asterisk denote significant estimates/comparisons (*P < 0.05, **P < 0.01, ***P < 0.001).

^a^Note that ecotype averages are indices calculated with values for all individuals, not arithmetic means of population values.

**Table 5.** Analysis results for number of sires per litter, female reproductive success, male reproductive success, and female size in *Thamnophis elegans* populations

Source of variation	Number of sires per litter (>58% sampled; N = 56)	Female reproductive success	Male reproductive success	Female size (SVL)
Ecotype				
Test statistic	$\chi_{1}^{2}$ = 0.024	*F*_1,49_ = 3.31	*F*_1,62_ = 3.53	***F***_**1,50**_ = **94.26**
*P*-value	0.88	0.075	0.065	<0.0001***
Litter size sampled				
Test statistic	$\chi_{1}^{2}$ = 1.76	—	—	—
*P*-value	0.19	—	—	—
Population (ecotype)				
Test statistic	$\chi_{1}^{2}$ = 3.63	***F***_**4,49**_ = **4.90**	*F*_4,62_ = 1.91	*F*_4,50_ = 0.87
*P*-value	0.46	0.0021**	0.12	0.49
Body size				
Test statistic	$\chi_{1}^{2}$ = 0.0038	***F***_**1,49**_ = **6.66**	—	—
*P*-value	0.95	0.013*	—	—

“—” indicates effect not included in model (see text for details). Asterisk denote significant factors (*P < 0.05, **P < 0.01, ***P < 0.001).

**Figure 3.**
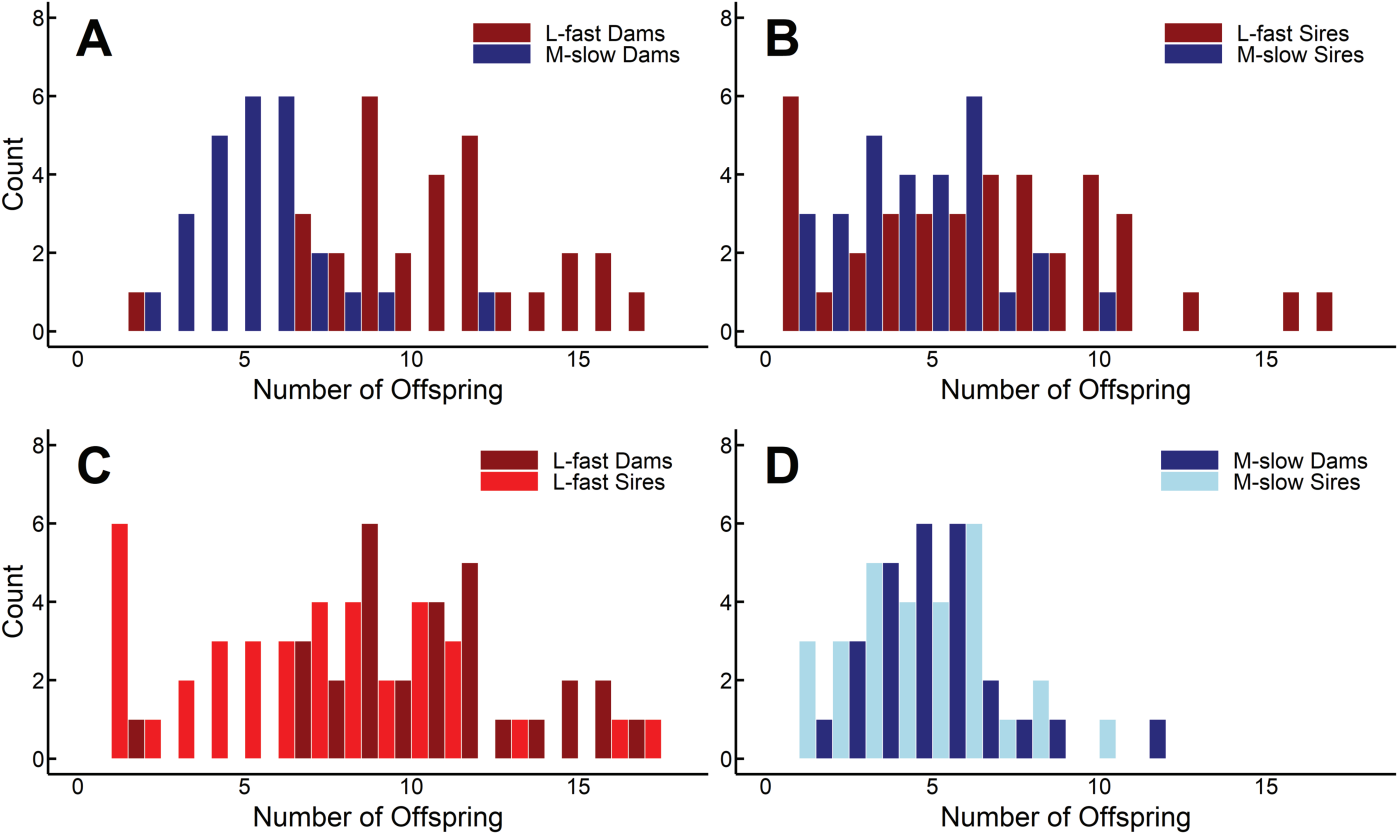
Distribution of reproductive success of sires and dams in wild populations of *Thamnophis elegans*: comparing ecotypes within each sex (A and B) and comparing sexes within each ecotype (C and D). For males, the number of offspring signifies the total number of offspring that can be attributed to a single male of all of the offspring sampled within a given population. For females, the number of offspring is her liveborn offspring. (A) Significant distribution differences between M-slow and L-fast dams (*P* < 0.0001); (B) significant distribution differences between M-slow and L-fast sires (*P* = 0.010). (C) Significant distribution differences between sexes within the L-fast ecotype (*P* = 0.001), whereas sexes did not differ in the M-slow ecotype (D).

Female reproductive success differed between ecotypes, with L-fast females having larger litters on average than M-slow litters (raw data quartiles: L-fast = 9, 11, 12, 17; M-slow: 4, 5, 6, 12). This is primarily due to body size differences, as ecotypes differed marginally in female reproductive success after correction for body size (LSmean ± SE: L-fast = 8.8 ± 0.6 offspring, M-slow = 6.8 ± 0.7, Table 5, Figure 3A; body size LS mean ± SE for M-slow females 473 ± 8.2 mm and L-fast females 590 ± 8.8 mm; Table 5; see also Bronikowski and Arnold 1999 and Sparkman et al. 2007). Finally, litter size differed among populations, and it is noteworthy that female reproductive success for one L-fast population was equal to that of one of the M-slow populations (Table 4). Similar to females, reproductive success of males, measured as the total number of offspring each male sired, contrasted between ecotypes with a trend for higher success in the L-fast ecotype (LSmean ± SE: 6.6 ± 0.8 offspring per male) compared with the M-slow ecotype (4.5 ± 0.8 offspring per male; Table 5, Figure 3B).

### Estimates of Reproductive Skew

When we compared the distributions of reproductive success within each sex between ecotypes, we found that the L-fast males and females had significantly right-shifted distributions (i.e., more individuals exhibiting high reproductive success) compared with M-slow males and females (Kolmogorov–Smirnov test, males: *D* = 0.40, *P* = 0.010; females: *D* = 0.77, *P* < 0.0001; Figure 3A and B), which is in agreement with the trend for differences in mean reproductive success within each sex. Within ecotypes, the distribution in reproductive success differed between males and females in L-fast populations (Kolmogorov–Smirnov test, L-fast males and females: *D* = 0.47, *P* = 0.001) but not in M-slow populations (*D* = 0.23, *P* = 0.49; Figure 3C and D). At the ecotype level, the Index of Variability (I-V), which tests the variance among individuals of the same sex within each population, was inflated above one in males from both ecotypes, but not in females (Table 4). This indicates that males of both ecotypes had greater among-individual variation in reproductive success than if reproduction followed a Poisson distribution, with L-fast males demonstrating much greater skew than M-slow males. In females, reproductive success was more evenly spread across individuals. At the population level, the test for sire skew within litters indicated that 3 populations were significantly skewed (2 L-fast populations and 1 M-slow population), which means that within a given litter, some males fathered more offspring than others. However, the 2 ecotypes were not significantly different from one another in within-litter reproductive skew (Table 4).

## Discussion

We found that multiple paternity was common in all 6 sampled populations of both life-history ecotypes. Although L-fast females have larger litter sizes and reproduce more frequently than M-slow females, both the number of sires within a litter and the frequency of multiply-sired litters did not differ between ecotypes. Based on life-history and morphological differences between the ecotypes, we had predicted the L-fast ecotype to have a higher frequency of multiple paternity due to a positive association between litter size and the potential for more sires to contribute to a litter. However, the generally higher measures of reproductive skew among sires (both within and among litters in the L-fast ecotype) suggest that M-slow and L-fast individuals are using different tactics to achieve reproductive success. At this time, we cannot assess whether this is due to courtship behavior of males or receptivity behavior of females. Furthermore, our estimates of reproductive skew suggest the hypothesis that M-slow dams are actively engaging in reproductive strategies to increase the level of multiple paternity within litters. However, our permutation analysis suggests that the mean number of sires contributing to litters would not differ between ecotypes if litters sizes were equivalent. Both female strategy and litter size likely contribute to observed patterns of multiple paternity and reproductive skew between the ecotypes.

Differences in M-slow female mating strategy, including both pre- and postcopulatory mechanisms, may compensate for small litter sizes with the outcome of similar multiple paternity levels and number of sires per litters as seen in L-fast populations. This increase in multiple paternity in M-slow females could result from several non-mutually exclusive possibilities: increased mating events and decreased precopulatory female choice relative to L-fast females, increased intrasexual competition among males resulting in greater variance in male reproductive success (and therefore more males producing no offspring), increased cryptic female choice, or potential female bet-hedging in the stochastic habitats of the M-slow populations. Work in other systems demonstrates the potential role of the environment in shaping multiple paternity patterns. For example, the increased level of relative multiple paternity in M-slow populations could result from shorter breeding seasons in the cooler meadow habitats (e.g., in watersnakes: Prosser et al. 2002). On the contrary, the relatively high levels of multiple paternity in M-slow snakes are counter to studies in birds and fish that found more extrapair matings in shorter-lived species and increased levels of multiple paternity in populations experiencing higher predation (Arnold and Owens 2002; Neff et al. 2008). This highlights the need to incorporate both life-history patterns and a characterization of environmental conditions in revealing the drivers of multiple paternity.

### Multiple Paternity in a Life-History Context

Previous studies on multiple paternity in garter snakes found positive relationships between litter size and number of sires within a litter (Garner et al. 2002; Garner and Larsen 2005), but this pattern is not universal in snakes (Blouin-Demers et al. 2005; Uller and Olsson 2008). In contrast to these previous studies in garter snakes, we found no association between the number of sires with either larger dams or larger litter size (Table 5). Furthermore, the results of our simulations show that differences in female reproduction between the ecotypes are due to difference in both strategy and litter size. Decreasing the litter size of L-fast dams does not affect the level of multiple paternity. That we did observe increased reproductive skew in both L-fast males and females compared to M-slow males and females suggests that the relationships among morphology, life-history, and multiple paternity is influenced by ecological or physiological variables (Uller and Olsson 2008). We speculate that mating with multiple males confers greater indirect benefits to females and reduces male reproductive skew in M-slow populations, though future work is needed to test these hypotheses directly. Previous work in reptiles points to fitness benefits for females with multiple mates, though the mechanisms conferring these benefits have yet to be thoroughly explored (Olsson et al. 1997; Madsen et al. 2002, 2005; Blouin-Demers et al. 2005). The long-lived females of the M-slow populations may enjoy any combination of possible benefits of multiple matings: “trading-up” (i.e., mating with a successive male after already mating, if the new male appears to be of higher quality); sperm competition (Parker 2020); cryptic female choice; fitness increases through the production of sons that achieve increased fitness (“sexy sons”); and bet-hedging (i.e., mating with several males in order to increase the genetic diversity of offspring; reviewed in Uller and Olsson 2008; Wapstra and Olsson 2014; Friesen et al. 2020). Bet-hedging, in particular, has been found to be a viable explanation for multiple paternity in either small or environmentally variable populations (Yasui, 1998, 2001; Calsbeek et al. 2007; Sarhan and Kokko, 2007; Makinen et al. 2007), such as the M-slow populations in this study (Miller et al. 2011). Additionally, there is some evidence for cryptic female choice in garter snakes (Friesen, Kerns, et al. 2014). However, female garter snakes can store sperm for at least a year (Friesen, Mason, et al. 2014), which means that apparent female choice within a season is not discernable from among-season sperm storage and usage (Friesen, Kerns, et al. 2014; Friesen et al. 2016). Moreover, males deposit significantly fewer sperm with successive matings (Friesen, Uhrig, et al. 2014). Future work in garter snakes can be directed toward specific tests of these contrasting, but not exclusive, hypotheses of sperm competition versus female choice.

### Multiple Paternity in a Demographic Context

In the absence of strong evidence to show that females benefit from mating multiple times, levels of mating may simply result from population density and encounter rate (Uller and Olsson 2008, Wells et al. 2017). Although we did not have a reliable method for determining the population density or encounter rates at the time of mating, we were able to calculate operational sex ratios during the active season to estimate one aspect of population demographics. In both ecotypes, more adult females than males were observed over 4 years of capture/mark/recapture fieldwork, but in L-fast populations, this female bias was far more pronounced. The average male to female operational sex ratio was 0.55 in L-fast populations and 0.71 in M-slow populations, indicating a strong bias toward female captures (Table 4). In 2006, the year in which the gravid females in this study were collected, our estimates are similar to the longer-term averages (L-fast OSR = 0.45, M-slow OSR = 0.65). These estimates of sex bias in the adult populations may result from males suffering higher mortality rates associated with more frequent long-distance movements, as identified in other garter snake systems (e.g., Bonnet et al. 1999; Shine et al. 2001a). The female-biased sex ratio may also be compensated for by the temporal distribution of mates in the garter snake mating system, which provides a strongly male-biased sex ratio during the primary time of mating. For example, in many garter snake populations, males congregate around hibernacula and compete by scramble competition for emerging females. The males stay at the hibernaculum for up to several weeks, while the females disperse in a few days (e.g., Shine et al. 2001b).

### Conclusions and Future Directions

Our findings suggest that other factors beyond operational sex ratios and male/female encounter rates might underlie the unexpected equivalence in degree of multiple paternity found in these divergent populations of garter snakes. Sperm competition, female reproductive strategies (i.e., bet-hedging, cryptic female choice), behavioral differences in males from L-fast populations (Gangloff et al. 2017), or a combination of any of these mechanisms might offer a more complete representation of the complex interactions that contribute to multiple paternity in these populations. In a sex-specific transcriptome scan of these populations, loci for proteins involved in sperm/egg interactions were identified as being highly variable and potentially under diversifying selection, which could suggest on-going sperm competition (Schwartz et al. 2010). Thus, sperm competition (i.e., sperm morphology, energetics, and swimming performance) is a potential determinant of the skew observed within litters, as has been suggested in other studies (Olsson and Madsen 1998; Friesen, Kerns, et al. 2014; Friesen, Mason, et al. 2014). Future studies utilizing a genome-wide approach could also point to fruitful candidate loci for discerning mechanisms of intrasexual competition and targets of selection in males (see also Levine et al. 2015). Controlled matings in a laboratory setting might also be helpful in determining if mating order has any implications for the reproductive success of males and if male quality (i.e., body condition, sperm quality) has any impact on the survival of the offspring they sire. Additionally, observations of mating behaviors in the field might provide insight into whether females are exerting choice in the form of mating preferences, or if male behavioral aspects (e.g., predator avoidance, mating coercion) are more important in determining the number of matings occurring in populations (e.g., Clark et al. 2014; Lind et al. 2016). Given the prevalence of observed multiple paternity and the known life-history differences among these populations, this system of garter snakes provides a model system to explore these important questions.

## Supplementary Material

esab043_suppl_Supplementary_Material

## Data Accessibility

Data are available through FigShare data repository at https://doi.org/10.25380/iastate.15070029.

## References

[CIT0001] Addis EA , Gangloff EJ, Palacios MG, Carr KE, Bronikowski AM. 2017. Merging the “morphology–performance–fitness” paradigm and life-history theory in the eagle lake garter snake research project. Integr Comp Biol. 57(2):423–435. 28859418 10.1093/icb/icx079

[CIT0002] Araki H , Robin S W, William R A, Becky C, Michael S B. 2007. Effective population size of steelhead trout: influence of variance in reproductive success, hatchery programs, and genetic compensation between life-history forms. Mol Ecol. 16(5):953–966. 17305853 10.1111/j.1365-294X.2006.03206.x

[CIT0003] Arnold KE , Owens IPF. 2002. Extra-pair paternity and egg dumping in birds: life history, parental care and the risk of retaliation. Proc R Soc Lond B Biol Sci. 269(1497):1263–1269. 10.1098/rspb.2002.2013 PMC1691019 12065043

[CIT0004] Arnold SJ , Peterson CR. 2002. A model for optimal reaction norms: the case of the pregnant garter snake and her temperature-sensitive embryos. Am Nat. 160(3):306–316. 18707441 10.1086/341522

[CIT0005] Becher SA , Magurran AE. 2004. Multiple mating and reproductive skew in Trinidadian guppies. Proc R Soc Biol Sci. 271:1009–1014. 10.1098/rspb.2004.2701 PMC1691690 15293853

[CIT0006] Blouin-Demers G , Gibbs HL, Weatherhead PJ. 2005. Genetic evidence for sexual selection in black ratsnakes, *Elaphe obsoleta*. Anim Behav. 69(1):225–234.

[CIT0007] Bolstein D , White RL, Skolnick M, Davis RW. 1980. Construction of a genetic linkage map in man using resticrition fragment length polymorphisms. Am J Hum Genet. 32:314–331. 6247908 PMC1686077

[CIT0008] Bonnet X , Guy N, Shine R. 1999. The dangers of leaving home: dispersal and mortality in snakes. Biol Conserv. 89(1):39–50.

[CIT0009] Boutin-Ganache I , Raposo M, Raymond M, Deschepper CF. 2001. M13-tailed primers improve the readability and usability of microsatellite analyses performed with two different allele-sizing methods. BioTechniques. 31(1):24–6, 28. 11464515

[CIT0010] Bronikowski AM , Arnold SJ. 1999. The evolutionary ecology of life history variation in the garter snake *Thamnophis elegans*. Ecology. 80(7):2314–2325. 10.1890/08-0850.1 19341142

[CIT0011] Calsbeek R , Bonneaud C, Prabhu S, Manoukis N, Smith TB. 2007. Multiple paternity and sperm storage lead to increased genetic diversity in *Anolis* lizards. Evol Ecol Res. 9(3):495–503.

[CIT0012] Charlesworth B . 2009. Fundamental concepts in genetics: effective population size and patterns of molecular evolution and variation. Nat Rev Genet. 10(3):195–205. 19204717 10.1038/nrg2526

[CIT0013] Clark RW , Schuett GW, Repp RA, Amarello M, Smith CF, Herrmann HW. 2014. Mating systems, reproductive success, and sexual selection in secretive species: a case study of the western diamond-backed rattlesnake, *Crotalus atrox*. PLoS One. 9(3):e90616. 24598810 10.1371/journal.pone.0090616 PMC3944027

[CIT0014] Clutton-Brock TH . 1989. Review lecture: mammalian mating systems. Proc R Soc Lond B Biol Sci. 236(1285):339–372. 2567517 10.1098/rspb.1989.0027

[CIT0015] Croshaw DA , Pechmann JHK, Glenn TC. 2017. Multiple paternity benefits female marbled salamanders by increasing survival of progeny to metamorphosis. Ethology. 123: 307–315.

[CIT0016] Excoffier L , Lavel G, Schneider S. 2005. *Arlequin* v. 3.0: An integrated software package for population genetics data analysis. Evol Bioinform Online. 1:47–50. PMC2658868 19325852

[CIT0017] Friesen CR , Kahrl AF, Olsson M. 2020. Sperm competition in squamate reptiles. Philos Trans R Soc B. 375: 20200079. 10.1098/rstb.2020.0079 PMC7661455 33070739

[CIT0018] Friesen CR , Kerns AR, Mason RT. 2014. Factors influencing paternity in multiply mated female red-sided garter snakes and the persistent use of sperm stored over winter. Behav Ecol Sociobiol. 68(9):1419–1430.

[CIT0019] Friesen CR , Mason RT, Arnold SJ, Estes S. 2014. Patterns of sperm use in two populations of red-sided garter snake (*Thamnophis sirtalis parietalis*) with long-term female sperm storage. Can J Zool. 92(1):33–40.

[CIT0020] Friesen CR , Uhrig EJ, Mason RT. 2014. Females remate more frequently when mated with sperm-deficient males. J Exp Zool A Ecol Genet Physiol. 321: 603–609. 25366702 10.1002/jez.1892

[CIT0021] Friesen CR , Uhrig EJ, Mason RT, Brennen PLR. 2016. Female behaviour and the interaction of male and female genital traits mediate sperm transfer during mating. J Evol Biol. 29: 952–964 26809830 10.1111/jeb.12836

[CIT0022] Gangloff EJ , Chow M, Leos-Barajas V, Hynes S, Hobbs B, Sparkman AM. 2017. Integrating behaviour into the pace-of-life continuum: divergent levels of activity and information gathering in fast-and slow-living snakes. Behav Process. 142: 156–163. 10.1016/j.beproc.2017.06.006 28648696

[CIT0023] Gangloff EJ , Schwartz TS, Klabacka R, Huebschman N, Liu AY, Bronikowski AM. 2020. Mitochondria as central characters in a complex narrative: linking genomics, energetics, pace-of-life, and aging in natural populations of garter snakes. Exp Gerontol. 137:110967. 32387125 10.1016/j.exger.2020.110967

[CIT0024] Gangloff EJ , Vleck D, Bronikowski AM. 2015. Developmental and immediate thermal environments shape energetic trade-offs, growth efficiency, and metabolic rate in divergent life-history ecotypes of the garter snake *Thamnophis elegans*. Physiol Biochem Zool. 88(5):550–563. 26658251 10.1086/682239

[CIT0025] Garner TWJ , Gregory PT, Mccracken GF, Burghardt GM, Koop BF, Mclain SE, Nelson RJ. 2002. Geographic variation of multiple paternity in the common garter snake (*Thamnophis sirtalis*). Copeia. 2002(1):15–23.

[CIT0026] Garner TWJ , Larsen KW. 2005. Multiple paternity in the western terrestial garter snake, *Thamnophis elegans*. Can J Zool. 83:656–663.

[CIT0027] Garner TWJ , Pearman PB, Gregory PT, Tomio G, Wischniowski SG, Hosken DJ. 2004. Microsatellite markers developed from *Thamnophis elegans* and *Thamnophis sirtalis* and their utility in three species of garter snakes. Mol Ecol Notes. 4(3):369–371.

[CIT0028] Gerlach NM , Mcglothlin JW, Parker PG, Ketterson ED. 2012. Promiscuous mating produces offspring with higher lifetime fitness. Proc R Soc B. 279(1730):860–866. 10.1098/rspb.2011.1547 PMC3259935 21881136

[CIT0029] Glaudas X , Rice SE, Clark RW, Alexander GJ. 2020. The intensity of sexual selection, body size and reproductive success in a mating system with male–male combat: is bigger better? Oikos. 129: 998–1011.

[CIT0030] Goudet J . 2001. FSTAT, a program to estimate and test gene diversities and fixation indices (version 2.9.3). Available from: http://www.unil/ch/izea/softwares/fstat.html.

[CIT0031] Gregory PT , Skebo KM. 1998. Trade-offs between reproductive traits and the influence of food intake during pregnancy in the garter snake, *Thamnophis elegans*. Am Nat. 151(5):477–486. 18811321 10.1086/286134

[CIT0032] Hoelzel AR . 1999. Impact of population bottlenecks on genetic variation and the importance of life-history; a case study of the northern elephant seal. Biol J Linn Soc. 68(1–2):23–39.

[CIT0033] Jellen BC , Aldridge RD. 2011. Paternity patterns. In R. D. Aldridge, D. M. Sever, editors. Reproductive biology and phylogeny of snakes, vol. 9. Enfield (NH); Boca Raton (FL): Science Publishers; Marketed and distributed by CRC Press. p. 619–644.

[CIT0034] Jennions MD , Petrie M. 2000. Why do females mate multiply? A review of the genetic benefits. Biol Rev. 75(1):21–64. 10740892 10.1017/s0006323199005423

[CIT0035] Kalinowski ST , Taper ML, Marshall TC. 2007 Revising how the computer program cervus accommodates genotyping error increases success in paternity assignment. Mol Ecol. 16(5):1099–1106. 17305863 10.1111/j.1365-294X.2007.03089.x

[CIT0036] Levine BA , Smith CF, Schuett GW, Douglas MR, Davis MA, Douglas, ME. 2015. Bateman-Trivers in the 21st century: sexual selection in a North American pitviper. Biol J Linn Soc. 114: 436–445.

[CIT0037] Lind CM , Flack B, Rhoads DD, Beaupre SJ. 2016. The mating system and reproductive life history of female Timber Rattlesnakes in northwestern Arkansas. Copeia. 104(2):518–528.

[CIT0038] Luo J , Sanetra M, Schartl M, Meyer A. 2005. Strong reproductive skew among males in the multiply mated swordtail *Xiphophorus multilineatus* (Teleostei). J Hered. 96(4):346–355. 15743903 10.1093/jhered/esi042

[CIT0039] Madsen T , Shine R. 1999. The adjustment of reproductive threshold to prey abundance in a capital breeder. J Anim Ecol. 68(3):571–580.

[CIT0040] Madsen T , Shine R, Loman J, Hakansson T. 2002. Why do female adders copulate so frequently? Nature. 355:440–441.

[CIT0041] Madsen T , Ujvari B, Olsson M, Shine R. 2005. Paternal alleles enhance female reproductive success in tropical pythons. Mol Ecol. 14(6):1783–1787. 15836649 10.1111/j.1365-294X.2005.02505.x

[CIT0042] Makinen T , Panova M, Andre C. 2007. High levels of multiple paternity in *Littorina saxatilis*: hedging the bets? J Hered. 98(7):705–711. 18056922 10.1093/jhered/esm097

[CIT0043] Manier MK , Arnold SJ. 2005. Population genetic analysis identifies source-sink dynamics for two sympatric garter snake species *(Thamnophis elegans and Thamnophis sirtalis)*. Mol Ecol. 14(13):3965–3976. 16262852 10.1111/j.1365-294X.2005.02734.x

[CIT0045] Manier MK , Seyler CM, Arnold SJ. 2007. Adaptive divergence within and between ecotypes of the terrestrial garter snake, *Thamnophis elegans*, assessed with FST-QST comparisons. J Evol Biol. 20(5):1705–1719. 17714288 10.1111/j.1420-9101.2007.01401.x

[CIT0046] Massey FJ . 1951. The Kolmogorov–Smirnov test for goodness of fit. J Am Stat Assoc. 46(253):68–78.

[CIT0047] Mccracken GF , Burghardt GM, Houts SE. 1999. Microsatellite markers and multiple paternity in the garter snake *Thamnophis sirtalis*. Mol Ecol. 8(9):1475–1479. 10564453 10.1046/j.1365-294x.1999.00720.x

[CIT0048] Meister B , Ursenbacher S, Baur B. 2012. Frequency of multiple paternity in the grass snake (*Natrix natrix*). Amphib-Reptilia. 33: 308–312.

[CIT0049] Miller DA , Clark WR, Arnold SJ, Bronikowski AM. 2011. Stochastic population dynamics in populations of western terrestrial garter snakes with divergent life histories. Ecology. 92(8):1658–1671. 21905432 10.1890/10-1438.1

[CIT0050] Neff BD , Pitchar TE, Ramnarine IW. 2008. Inter-population variation in multiple paternity and reproductive skew in the guppy. Mol Ecol. 17(12):2975–2984. 18494765 10.1111/j.1365-294X.2008.03816.x

[CIT0051] Nei M . 1987. Molecular evolutionary genetics. New York: Columbia University Press.

[CIT0052] O’donnell RP , Arnold SJ. 2005. Evidence for selection on thermoregulation: effects of temperature on embryo mortality in the garter snake *Thamnophis elegans*. Copeia. 2005(4):930–934.

[CIT0053] Olsson M , Gullberg A, Tegelstrom H. 1997. Determinants of breeding dispersal in the sand lizard, *Lacerta agilis*, (Reptilia, Squamata). Biol J Linn Soc 60(2):243–256.

[CIT0054] Olsson M , Madsen T. 1998. Sexual selection and sperm competition in reptiles. In: Birkhead TR, Møller A, editors. Sperm competition and sexual selection. San Diego (CA): Academic Press. p. 503–577

[CIT0089] Palacios MG , Bronikowski AM. 2017. Immune variation during pregnancy suggests immune component-specific costs of reproduction in a viviparous snake with disparate life-history strategies. J Exp Zool A: Ecol Integr Physiol. 327:513–522. 29356424 10.1002/jez.2137

[CIT0055] Palacios MG , Sparkman AM, Bronikowski AM. 2012. Corticosterone and pace of life in two life-history ecotypes of the garter snake *Thamnophis elegans*. Gen Comp Endocrinol. 175(3):443–448. 22178432 10.1016/j.ygcen.2011.11.042

[CIT0056] Parker GA . 2020. Conceptual developments in sperm competition: a very brief synopsis. Philos Trans R Soc B. 375: 20200061 10.1098/rstb.2020.0061 PMC7661437 33070727

[CIT0057] Pearse DE , Anderson EC. 2009. Multiple paternity increases effective population size. Mol Ecol. 18(15):3124–3127. 19555411 10.1111/j.1365-294X.2009.04268.x

[CIT0058] Prosser MR , Gibbs HL, Weatherhead PJ. 1999. Microgeographic population genetic structure in the northern water snake, *Nerodia sipedon sipedon* detected using microsatellite DNA loci. Mol Ecol. 8(2):329–333. 10065548 10.1046/j.1365-294x.1999.00530.x

[CIT0059] Prosser MR , Weatherhead PJ, Gibbs HL, Brown GP. 2002. Genetic analysis of the mating system and opportunity for sexual selection in northern water snakes (*Nerodia sipedon*). Behav Ecol. 13(6):800–807.

[CIT0060] Raymond M , Rousset F. 1995. Population genetics software for exact tests and ecumenicism. J Hered. 86:248–249.

[CIT0061] Rice WR . 1989. Analyzing tables of statistical tests. Evolution. 43(1):223–225. 28568501 10.1111/j.1558-5646.1989.tb04220.x

[CIT0062] Rivas JA , Burghardt GM. 2005. Snake mating systems, behavior, and evolution: the revisionary implications of recent findings. J Comp Psychol. 119(4):447–454. 16366778 10.1037/0735-7036.119.4.447

[CIT0063] Robert KA , Bronikowski AM. 2010. Evolution of senescence in nature: physiological evolution in populations of garter snake with divergent life histories. Am Nat. 175(2):147–159. 20050804 10.1086/649595

[CIT0064] Roff D . 1992. The evolution of life histories: theory and analysis. New York: Chapman & Hall.

[CIT0065] Rossman DA , Ford NB, Siegel RA. 1986. The garter snakes: evolution and ecology. Norman (OK): University of Oklahoma Press.

[CIT0066] Sarhan A , Kokko H. 2007. Multiple mating in the Glanville fritillary butterfly: a case of within-generation bet hedging? Evolution. 61(3):606–616. 17348923 10.1111/j.1558-5646.2007.00053.x

[CIT0067] Schwartz TS , Bronikowski AM. 2013. Dissecting molecular stress networks: indentifying nodes of divergence between life-history phenotypes. Mol Ecol. 22(3):739–756. 22988821 10.1111/j.1365-294X.2012.05750.x

[CIT0092] Schwartz TS , Choi J-H, Tae H, Yang Y, Mockaitis K, Van Hemert JL, Proulx SR, Bronikowski AM. 2010. A garter snake transcriptome: pyrosequencing, *de novo* assembly, and sex-specific differences. BMC Genomics. 11:694. 21138572 10.1186/1471-2164-11-694 PMC3014983

[CIT0068] Schwarzkopf L , Shine R. 1991. Thermal biology of reproduction in viviparous skins, *Eulamprus tympanum*: why do gravid females back more? Oecologia. 88(4):562–569. 28312627 10.1007/BF00317720

[CIT0069] Seigel RA , Huggins MM, Ford NB. 1987. Reduction in locomotor ability as a cost of reproduction in gravid snakes. Oecologia. 73(4):481–485. 28311962 10.1007/BF00379404

[CIT0070] Shine R , Elphick MJ, Harlow PS, Moore IT, Lemaster MP, Mason RT. 2001a. Movements, mating, and dispersal of red-sided gartersnakes (*Thamnophis sirtalis parietalis*) from a communal den in Manitoba. Copeia. 2001(1):82–91.

[CIT0071] Shine R , Lemaster M, Moore I, Olsson M, Mason R. 2001b. Bumpus in the snake den: effects of sex, size, and body condition on mortality of red-sided garter snakes. Evolution. 55(3):598–604. 11327166 10.1554/0014-3820(2001)055[0598:bitsde]2.0.co;2

[CIT0072] Sparkman AM , Arnold SJ, Bronikowski AM. 2007. An empirical test of evolutionary theories for reproductive senescence and reproductive effort in the garter snake *Thamnophis elegans*. Proc R Soc B Biol Sci. 274(1612):943–950. 10.1098/rspb.2006.0072 PMC2141668 17251099

[CIT0073] Sparkman AM , Bronikowski AM, Billings JG, Von Borstel D, Arnold SJ. 2013. Avian predation and the evolution of life-histories in the garter snake *Thamnophis elegans*. Am Midl Nat. 171(1):66–85.

[CIT0074] Stearns SC . 1992. The evolution of life histories. Oxford: Oxford University Press.

[CIT0075] Stedman AL , Jaeger CP, Hileman ET, Jellen BC, Phillips CA, Swanson BJ, King RB. 2016. Mulitple paternity in three wild populations of Eastern Massasauga (*Sistrurus catenatus*). Herpetol Conserv Biol. 11(1):160–167.

[CIT0076] Sunnucks P , Hales DF. 1996. Numerous transposed sequences of mitochondrial cytochrome oxidase I-II in aphids of the genus *Sitobion* (Hemiptera: Aphididae). Mol Biol Evol. 13(3):510–524. 8742640 10.1093/oxfordjournals.molbev.a025612

[CIT0090] Taylor ML , Price TAR, Wedell N. 2014. Polyandry in nature: a global analysis. Trends Ecol Evol. 29(7):376–383. 24831458 10.1016/j.tree.2014.04.005

[CIT0077] Uller T , Olsson M. 2008. Multiple paternity in reptiles: patterns and processes. Mol Ecol. 17(11):2566–2580. 18452517 10.1111/j.1365-294X.2008.03772.x

[CIT0078] Van Dyke JU , Beaupre SJ. 2011. Bioenergetic components of reproductive effort in viviparous snakes: costs of vitellogenesis exceed costs of pregnancy. Comp Biochem Physiol A. 160(4):504–515. 10.1016/j.cbpa.2011.08.011 21884815

[CIT0079] Van Oosterhout C , Hutchinson WF, Wills DPM, Shipley P. 2004. Micro-checker: software for identifying and correcting genotyping errors in microsatellite data. Mol Ecol Notes. 4(3):535–538.

[CIT0080] Voris HK , Karns DR, Feldheim KA, Kechavarzi B, Rinehart M. 2008. Multiple paternity in the Oriental-Australian rear-fanged watersnakes (Homalopsidae). Herpetol Conserv Biol. 3(1):88–102.

[CIT0081] Wang JL . 2004 Sibship reconstruction from genetic data with typing errors. Genetics. 166(4):1963–1979. 15126412 10.1534/genetics.166.4.1963 PMC1470831

[CIT0091] Wapstra E , Olsson M. 2014. The evolution of polyandry and patterns of multiple paternity in lizards. In: Rheubert J, Siegel D, Trauth S, Jamieson B, editors. The reproductive evology and phylogeny of lizards and tuatara. Science Publishers.

[CIT0082] Wedell N , Kvarnemo C, Lessells CM, Tregenza T. 2006. Sexual conflict and life histories. Anim Behav. 71(5):999–1011.

[CIT0083] Wells CP , Tomalty KM, Floyd CH, Mcelreath MB, May BP, Van Vuren, DH. 2017. Determinants of multiple paternity in a fluctuating population of ground squirrels. Behav Ecol Sociobiol. 71: 42–55.

[CIT0084] Worthington AM , Jurenka RA, Kelly CD. 2015. Mating for male-derived prostaglandin: a functional explanation for the increased fecundity of mated female crickets? J Exp Biol. 218(Pt 17):2720–2727. 26113140 10.1242/jeb.121327

[CIT0085] Worthington AM , Kelly CD. 2016. Females gain survival benefits from immune-boosting ejaculates. Evolution. 70(4):928–933. 26920335 10.1111/evo.12890

[CIT0086] Wusterbarth TL , King RB, Duvall MR, Grayburn WS, Burghardt GM. 2010. Phylogenetically widespread multiple paternity in New World natricine snakes. Herpetol Conserv Biol. 5(1):86–93.

[CIT0087] Yasui Y . 1998. The “genetic benefits” of female multiple mating reconsidered. Trends Ecol Evol. 13(6):246–250. 21238286 10.1016/s0169-5347(98)01383-4

[CIT0088] Yasui Y . 2001. Female multiple mating as a genetic bet-hedging strategy when mate choice criteria are unreliable. Ecol Res. 16(4):605–616.

