# Supplementary material for: Multiple Paternity in Garter Snakes With Evolutionarily Divergent Life Histories
Source: J Hered. 2021 Aug 5;112(6):508–18. doi: 10.1093/jhered/esab043 (PMC8558580; doi:10.1093/jhered/esab043)

Table S1. Population genetic structure among *Thamnophis elegans* populations. Pairwise F_{ST} values (calculated using 7 loci) below diagonal. "NS" indicates values that were not significant value after sequential Bonferroni correction. 3-letter codes after Manier et al. 2005. L and M codes after Bronikowski and Arnold 2005. See Fig. S1 for locations.

	L1 GAL	L2 PIK	L3 WCT	L4 ELF	L5 STO	L7 MER	L8 CHR	M1 PAP	M2 NML	M3 MAH
L2	0.024 NS									
L3	0.002 NS	0.001 NS								
L4	0.027	0.019	0.000 NS							
L5	0.080	0.061	0.027 NS	0.051						
L7	0.029	0.039	0.007 NS	0.040	0.091					
L8	0.017	0.021	0.000 NS	0.035	0.086	0.005 NS				
M1	0.030	0.076	0.046	0.075	0.113	0.021	0.023			
M2	0.031	0.067	0.042	0.072	0.111	0.022	0.028	0.003 NS		
M3	0.036	0.065	0.039	0.069	0.102	0.024	0.032	0.008	0.007 NS	
M5 SUM	0.034	0.047	0.000 NS	0.039	0.074	0.042	0.016	0.053	0.045	0.053

Figure S1. Map of population locations of *Thamnophis elegans* around Eagle Lake in Lassen County, California, USA included in the calculation of Fst in Table S1. L and M designators refer to replicate L-fast and M-slow populations. Map created by Katie Fetting using ArcGIS 10.3 (ESRI, Redlands, CA).

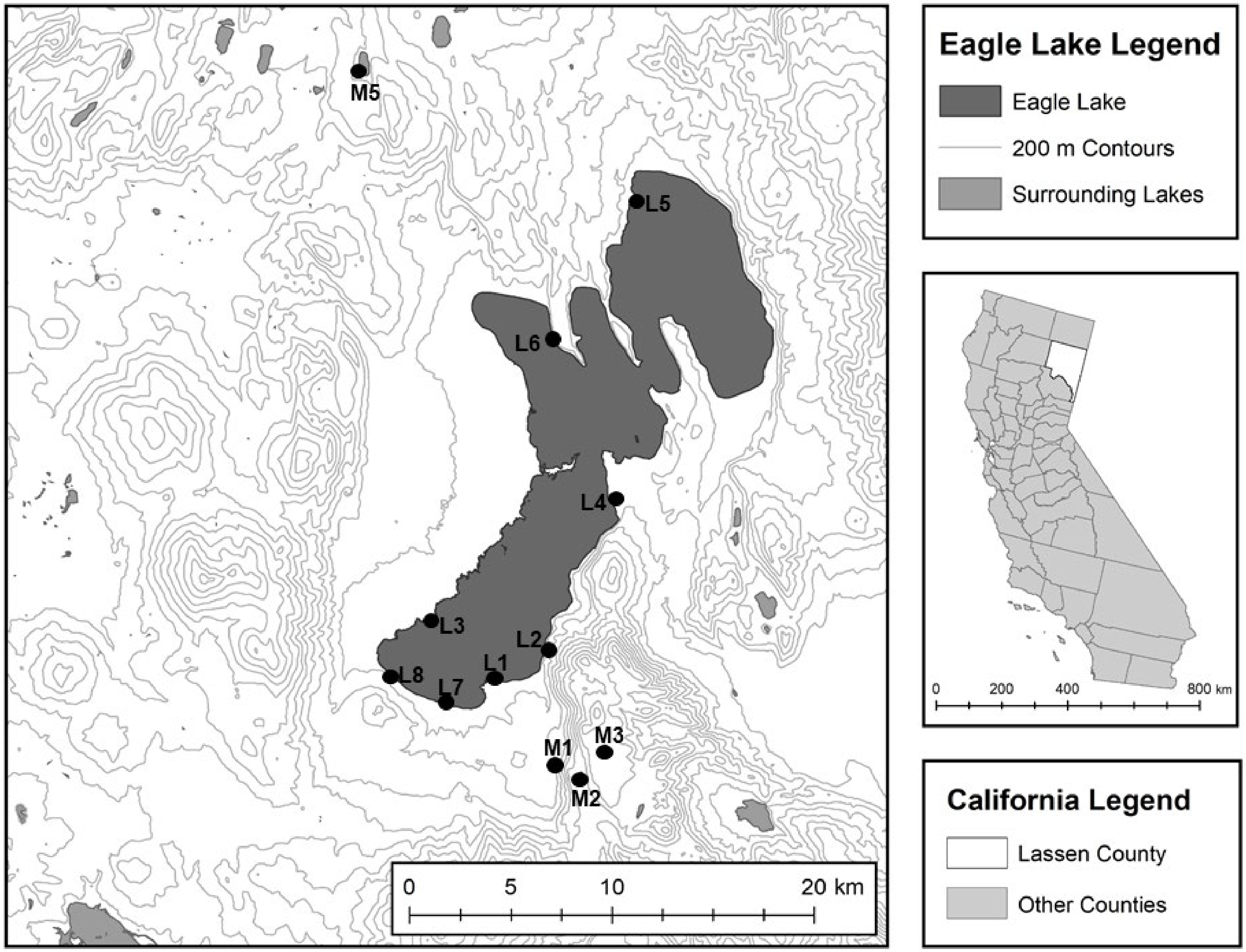

Supplement: esab043_suppl_Supplementary_Material [file esab043_suppl_supplementary_material.pdf]
